# Supplementary material for: Insulin Sensitivity Controls Activity of Pathogenic CD4+ T Cells in Rheumatoid Arthritis
Source: Cells. 2024 Dec 22;13(24):2124. doi: 10.3390/cells13242124 (PMC11674209; doi:10.3390/cells13242124)
Supplement: Supplementary file 1 [file cells-13-02124-s001.zip › cells-3371972-supplementary.pdf]

S1A

Clinical characteristics of RA patients and controls used in this study

	Experimental n=40	Healthy n=69	RA n=16	JAKi n=24	non-JAKi n=32
Age, y	46 ± 13	49.3 ± 16.9	59.9 ± 12.4 $	52.0 ± 12.9	64.8 ± 7.7 **
DD, y	NA	NA	3.3 ± 3.5	15.3 ± 10.4	12.2 ± 10.0
DAS28	NA	NA	3.53 ± 1.13	2.35 ± 0.96	2.78 ± 1.09
DAS28>3.2	NA	NA	9 (56%)	3 (9%)	9 (28%)
Hb, mg/L	NA	140 ± 12.3	136 ± 11.6	132 ± 6.7	139 ± 10.6
WBC, 10^9/L	NA	5.7 ± 1.8	6.1 ± 2.2	5.4 ± 1.7	6.1 ± 2.4
Platelets, 10^9/L	NA	267 ± 66	286 ± 84	308 ± 88	253 ± 57 *
CRP, mg/ml	NA	3.9± 8.9	7.7± 12.4	2.8 ± 5.4	2.9 ± 3.1

$, Healthy vs RA, p<0.05

* JAKi vs non-JAKi, p<0.05
** JAKi vs non-JAKi, p<0.01

S1B DNA content, gating strategy

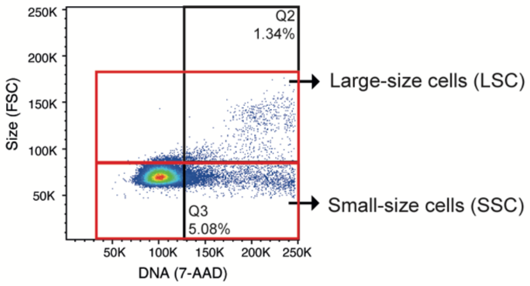

S1D

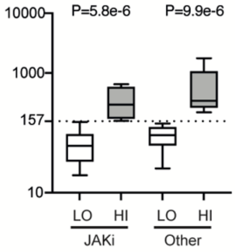

S1C. Primers used in qPCR

Target	Forward (5'to3')	Reverse (5'to3')	Manufacturer
ABL1	GCTGAGATACGAAGGGAGGG	TGGATAATGGAGCGTGGTGA	Sigma-Aldrich/Merck
BIRC5	GACCACCGCATCTCTACATTC	TGCTTTTTATGTTCCTCTATGGG	Sigma-Aldrich/Merck
FGR	GTGCCTACTCCCTGTCCATC	CACAGCCCGTCATTCACCT	Sigma-Aldrich/Merck
IFNG	TTTGGGTTCTCTTGGCTGTT	TCCGCTACATCTGAATGACCT	Sigma-Aldrich/Merck
INSR	AGATGACAACGAGGAGTGTGG	AGCCGTGTGACTTACAGATGG	Sigma-Aldrich/Merck
IRS1	GTTTCCAGAAGCAGCCAGAG	GGATTTGCTGAGGTCATTTAGG	Sigma-Aldrich/Merck
IRS2	CTTCTTGTCCCACCACTTGA	TGAAACAGTGCTGAGCGTCT	Sigma-Aldrich/Merck
PFKFB3	CCTACAACTTCTTCCGCCCC	CCGCAATTTGTCCCCCTTCT	Sigma-Aldrich/Merck
PIK3CG	GGCGACAGACACAATGACAA	GGGTTAGCACAAATGGCACT	Sigma-Aldrich/Merck
STAT5A	TGAAGACCCAGACCAAGTTTG	CCATCACGCAGCAGTTGTT	Sigma-Aldrich/Merck
SYK	CATCATCAGTCAGAAGCCTCAG	CGTAGGAGCCGTTGTTGTC	Sigma-Aldrich/Merck
TBET	CGCCAGGAAGTTTCATTTG	TTATGGAGGGACTGGAGCAC	Sigma-Aldrich/Merck
Reference gene		Assay number	
ACTB		qA-01-0104S	TATAA Biocenter

Supplementary Figure S1.

A. Clinical characteristics of RA patients and controls used in this study. Values are presented as mean+SD. $ Healthy vs RA, p<0.05, * JAKi vs non-JAKi, p<0.05, ** JAKi vs non-JAKi, p<0.01, by unpaired t-test. DD, disease duration; BMI, body mass index; DAS28, RA disease activity score by 28 joints; Hb, hemoglobin; WBC, white blood cell count; JAKi, Janus kinase inhibitors; y, years.

B. Gating strategy of small-size cells (SSC) and large-size cells (LSC) within CD4+ cells, by forward cell scatter and 7-aminoactinomycin D (7AAD) staining.

C. Sequences of primers used in qPCR.

D. Box plot of insulin levels within JAKi-treated (Hi, n=7, Lo, n=17) and non-JAKi treated (Other, Hi, n=5, Lo, n=27) RA patients. Plasma insulin levels 157 pmol/L was used to dichotomized patients in high (HI) and low (LO).

S2A

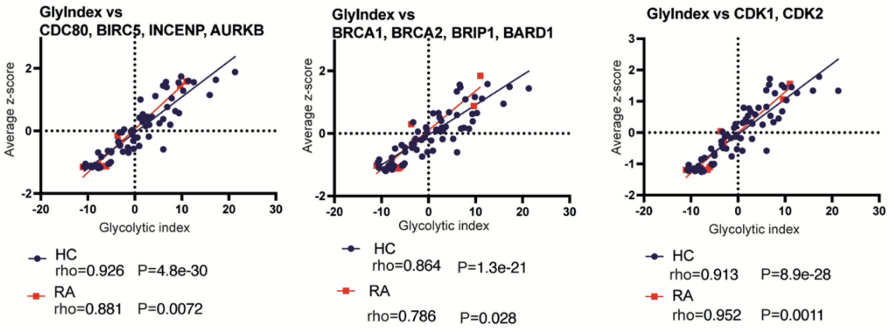

S2B

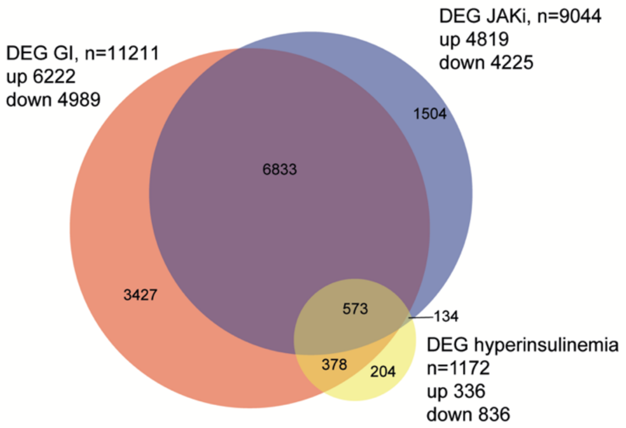

Supplementary Figure S2.

A. Scatterplot of Spearman's correlation between glycolytic index (GI) of CD4+ cells and genes involved in cell cycle control and DNA damage sensing *CDK1/2*, *BRCA1/2*, and chromosomal passenger complex proteins *AURKB*, *INCENP, CDCA8* and *BIRC5* of untreated RA patients (n=16, red dots) and matched HC (n=41, black dots). Solid lines indicate lineal regression curve.

B. Venn diagram of differentially expressed genes (DEG, expression basemean >10, nominal p-value<0.05) between CD4+ cells of HC with high and low glycolytic (GI-hi vs GI-Lo, red), of JAKi and non-JAKi treated RA patients (JAKi vs Other, blue), and of RA patients with hyperinsulinemia vs normal insulin levels (yellow). Plasma insulin levels 157 pmol/L was used to dichotomized patients.
